# Survey of Bacterial Chondronecrosis with Osteomyelitis Lesion Incidence in Broiler Farms in Kazakhstan Regions

**DOI:** 10.3390/ani16111584

**Published:** 2026-05-23

**Authors:** Anh Dang Trieu Do, Gulim Assetova, Andi Asnayanti, Aizhan Akhmetzhanova, Assel Zhexenayeva, Dauletbek Muratbayev, Dilora Senkebayeva, Bakytzhan Bolkenov, Adnan Alrubaye

**Affiliations:** 1 Cell and Molecular Biology Program, University of Arkansas, Fayetteville, AR 72701, USA; 2 The Department of Veterinary, Shakarim University, Semey 071400, Kazakhstan; assetova_gulim@shakarim.kz (G.A.); a.akhmetzhanova@shakarim.kz (A.A.); a.zheksenaeva@shakarim.kz (A.Z.); d.muratbayev@shakarim.kz (D.M.); b.bolkenov@shakarim.kz (B.B.); 3 Department of Poultry Science, University of Arkansas, Fayetteville, AR 72701, USA; aasnayan@uark.edu; 4 NJSC “National Agrarian Scientific and Educational Center”, Astana 010000, Kazakhstan; d.senkebayeva@nasec.kz

**Keywords:** poultry, broiler, lameness, BCO, Kazakhstan

## Abstract

Lameness associated with bacterial chondronecrosis with osteomyelitis (BCO) continues to be an important topic of great interest to global poultry production. Caused by bacterial infection of susceptible necrotic bone tissue, the disease severely affects animal health, welfare, and productivity, leading to significant economic losses annually. In recent years, the Kazakhstan poultry industry has enjoyed significant investment and strong growth, with goals of self-sufficiency within the decade. However, there remains a significant knowledge gap in poultry research in the nation, especially regarding the topic of BCO lameness. As such, this study aims to provide a preliminary evaluation of BCO lesion prevalence in different geographical regions of the country―namely Abai, Almaty, and Akmola. In each region, about 200 broilers from local poultry farms were procured, humanely euthanized, and necropsied to evaluate prevalence of femoral and tibial lesions commonly associated with BCO lameness. On average, most broilers had no damage to femoral head (78.17%) followed by femoral head separation (FHS, 11.94%), while the tibial head saw ubiquitous degrees of damage ranging from severe (71.42%) to observable (23.06%). These findings signify potential underlying issues connected to BCO lameness that will necessitate early management and intervention measures to prevent future spread of the disease.

## Simple Summary

Lameness from bacterial bone infection is an important issue affecting global poultry production, negatively impacting animal welfare and productivity. Despite this, there is a significant knowledge gap and documentation of this disease in many regions of the world, such as the Republic of Kazakhstan. As there are significant plans to improve the poultry industry in the region within the next decade, a survey of lesion prevalence commonly associated with bacterial lameness constitutes a significant contribution to robust and sustainable Kazakhstan poultry production. The results from this study will provide valuable insights for producers and researchers in the region in future establishment of effective management and prevention strategies against this disease.

## 1. Introduction

In recent years, the global poultry industry continues to lead among livestock species in the agriculture sector in the production of profitable, safe, and high-quality animal protein [1,2]. Owing to intense genetic selection within the past seven decades, the modern poultry has quadrupled in body weight while consuming half as much feed in six weeks compared to the ideal 1950s bird [3]. Despite this, serious health issues negatively impacting the animals continue to mire the industry annually, driving bird health, welfare, and industry bottom lines downward. Among them, infectious bacterial chondronecrosis with osteomyelitis (BCO) lameness remains as one of the top causes of poultry lameness and a “hot button” that has not yet been effectively addressed. Initiated by the broiler’s rapid weight gain and stress during the intensive production cycle, susceptible bones and joints are mechanically damaged and become prime breeding grounds for bacterial infections, further necrosis, and eventual lameness that can be spread within flocks [4,5,6]. While research of preventive and mitigatory strategies against the disease continues to be an important topic, scholars in the field have identified several effective management approaches ranging from early inclusion of glycosidic additives [7,8], probiotic application [9], and vaccine development [10,11]―though much work and industrial involvement remains, especially within an interspecies context where bacterial lameness pathology shares many similarities regardless of the target host [12,13,14].

Within the past decade, the Republic of Kazakhstan’s poultry industry has received increased attention and significant investment to address rising consumer demands. After suffering a drastic decrease in production volumes between 1990 and 2012 [15], the Kazakh government has devised detailed plans toward self-sufficiency in broiler meat production―which has traditionally been reliant on imports from neighboring regions [15,16]. A correspondent report in 2025 has reported an expansive structuring plan for at least 29 poultry farms, capable of producing 220,000 tonnes of broiler meat annually, before 2027 [17]. At the end of 2025, poultry farming in Kazakhstan showed significant growth in key indicators compared to the previous year. While at the beginning of 2025 the total poultry population in the country was 45,990,901, by the beginning of 2026 this figure had grown to 49,115,730, reflecting a steady trend of herd expansion of 6.8%. This growth is ensured both by the development of large industrial enterprises and the expansion of farm capacities. According to the National Statistics Bureau of the Agency for Strategic Planning and Reforms of the Republic of Kazakhstan, the slaughter weight amounted to 27,843.52 tons. The largest volumes of poultry slaughter are concentrated in several regions, such as Akmola region―7527.6 tons, Almaty region―7305.51 tons, East Kazakhstan region―4715.12 tons, and Abai region―1720.4 tons. These regions account for the bulk of industrial poultry meat production in the country [18]. By 2030, the country intends to double its exports of livestock products, including poultry meat. To this end, there are plans to expand the preferential lending program with a 5% interest rate subsidy from the state for poultry farms. One of the priorities is to enter the EU market, as well as to implement previously announced plans to export to China.

However, the current poultry health landscape in various Kazakh regions remain nebulous, with a 2025 study investigating poultry epidemiology citing frequent recurrences of serious infectious diseases, such as high-pathology avian influenza (HPAI), Newcastle disease virus (NDV), and widespread exposure to *Mycoplasma* spp., from deficient and inconsistent vaccination protocols, as well as inadequate biosecurity upkeep and management plans―particularly in small local farms [19]. The fragmented structure of the poultry sector, where industrial operations coexist with numerous small private holdings, further complicates disease control and surveillance efforts. In addition, Kazakhstan’s vast geographical territory and climatic variability pose logistical challenges for uniform veterinary oversight and rapid outbreak response. Cross-border trade and migratory bird pathways also increase the risk of transboundary disease introduction, particularly for avian influenza. While large commercial farms generally maintain structured biosecurity and vaccination programs, compliance gaps in smaller enterprises contribute to recurrent infection cycles and potential economic losses. Compliance with international food safety standards and animal health requirements, including the production of disease-free products, plays a key role in strengthening export potential, ensuring the trust of foreign partners and opening up access to new markets [20].

Overall, there also remains a critical knowledge gap in current literature regarding various aspects of Kazakh poultry research―such as nutrition and feed safety, antimicrobial resistance monitoring, and sustainable health management strategies―compared to other global regions with a strong poultry sector, which highlights a crucial weak point that may negatively impact its future. Strengthening epidemiological surveillance, harmonizing vaccination strategies, enhancing farm-level biosecurity training, and expanding research capacity in poultry nutrition and health are therefore essential steps toward improving resilience and sustainability within Kazakhstan’s poultry industry. Leveraging these factors, this surveillance study of BCO lesion prevalence in several important poultry-producing regions in Kazakhstan―namely Abai, Almaty, and Akmola―signifies an important first step in the investigation of this highly relevant poultry disease in the nation. To our knowledge, this is the first research project of this nature in the region. The early insights gleaned here are expected to contribute significantly to future research prospects of the Kazakh poultry industry, toward improvement of broiler welfare and sustainable production.

## 2. Materials and Methods

### 2.1. Animal Use Statement

The animal study protocol was approved by the Ethics Commission of Shakarim University (Protocol No. SU-EC-2024-003, approved on 24 October 2024). All procedures were performed in accordance with institutional and national guidelines.

### 2.2. Survey Design

**Figure 1:**
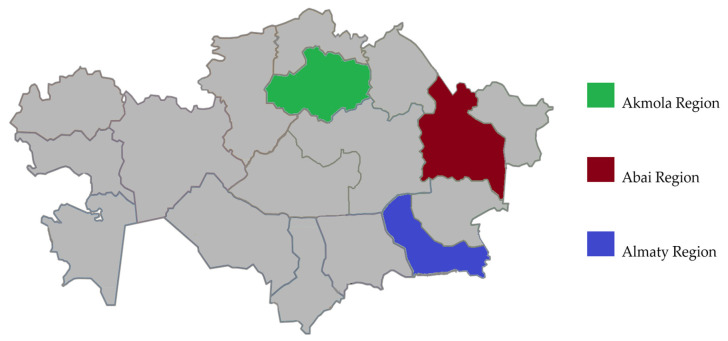
Map of BCO survey locations in the Abai, Almaty, and Akmola regions of Kazakhstan.

The survey took place in several regions of Kazakhstan spanning from November 2025 to January 2026, which are visualized in Figure 1.

Originally, approximately 200 broilers were intended for procurement from farms local to each surveyed region. Due to sourcing difficulties from the first surveyed region (Abai), broilers were procured from multiple farms with bird age ranging from 22 d to 60 d of age, several of which are unequal in size, totalling 207 surveyed animals in this region. To account for the influence of age in interregional comparisons, the data were stratified by age group, and the birds’ age was included as a covariate in the multivariate statistical models, thereby enabling a more accurate interpretation of the prevalence of lesions at various sampling sites. The number of birds surveyed in the Akmola and Almaty regions remained as planned (*n* = 200 per region). The total number of birds surveyed in all regions is 607 (*n* = 607). As the study was purely observational for surveying purposes, no control population was assigned.

Table 1 further details additional information pertaining to surveyed regions and animals.

Birds were maintained under routine farm conditions at each surveyed location, predominantly under floor housing on litter. In the first location in Abai (same sampling region hereby referred to as Abai-1 and Abai-2, respectively) birds were kept on small household-type farms; chicks were brooded up to 14 days of age under a temperature-controlled brooding regimen and were subsequently transferred to floor housing on litter (sawdust and wood shavings). During the floor-housing period, ambient temperatures were generally below recommended levels. Ventilation relied on forced exhaust, and lighting followed standard farm practice. Stocking density was approximately 10–12 birds/m^2^. Underfloor heating was available only in Abai-1, which was characterized by lower indoor relative humidity (~35–45%), whereas Abai-2 exhibited higher humidity (~55–70%) with visibly wetter litter. Similarly, elevated humidity and wet litter conditions were observed at the Almaty site(s), comparable to those recorded at Abai-2. In Astana, birds were housed on an industrial-type farm where zoohygienic conditions were more strictly controlled and met minimum industry requirements. Birds had ad libitum access to feed and clean water. Feeding followed a standard three-phase program (starter, grower, and finisher). At Abai-2, birds received a commercial complete compound feed without additional supplements. At Abai-1 and Almaty site(s), birds were fed commercial compound feed; however, cracked corn was additionally provided during the finisher phase. In Astana, birds received a farm-produced complete compound feed (on-site feed mill) within the starter–grower–finisher program.

### 2.3. Sampling Parameters

#### 2.3.1. Weight

All sampled birds were humanely euthanized via carbon dioxide gas inhalation, after which their individual body weight was recorded in grams using a digital table scale.

#### 2.3.2. Evaluation of Femoral Head and Tibial Head Lesions

**Figure 2:**
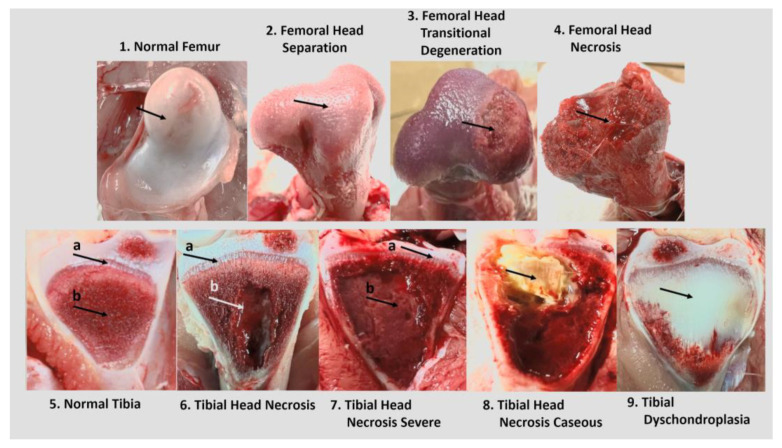
Reproduced with permission from Do et al. [9]. Examples of BCO lesion severity categories for diagnosis. Arrows provided to indicate hallmark characteristics: 1. Normal proximal femoral head state with intact epiphyseal articular cartilage; 2. proximal femoral epiphysis surface separated from cartilage that remains in the acetabulum; 3. separated proximal femoral epiphysis with varying degrees of damage (moderate lesion here with fibrinonecrotic exudate); 4. extreme damage to fracture of weakened proximal femoral epiphysis and physis upon disarticulation of the femur; 5. normal state of proximal tibia with clearly defined physeal growth plate (a) and firm cancellous bone (b); 6. necrotic state of the proximal tibia, still with clearly defined physeal growth plate (a) but damaged cancellous bone, replaced with a necrotic void of various sizes (b); 7. severe necrotic state of the proximal tibia, with physeal growth plate (a) encroached upon by large necrotic void (b); 8. necrotic state of the proximal tibia with additional caseous exudate, marking bacterial infiltration region; 9. proximal tibial head afflicted with tibial dyschondroplasia, marking abnormally large region of cartilage instead of cancellous bone.

Weighed birds were further necropsied to evaluate and record presence of common BCO lesions in their proximal femoral and tibial heads per routine protocol as previously published [6]. Briefly, both left and right proximal femora of each bird was dislocated from the acetabulum to assess the state of the cartilage cap, and/or the underlying epiphysis surface. Following femoral evaluation, the head of both left and right tibiae were exposed and cleanly sliced through at an angle with a sharp blade, after which the state of cancellous bone tissue was examined. Figure 2 further details classification of BCO lesion severity as per Do et al. [9].

In order to further investigate the possible relationship between bird body weight and development of BCO lesion, a scoring system was applied to femoral and tibial lesions (including tibial dyschondroplasia) as follows in Table 2.

A final ordinated score (“Body Score”) was calculated for each individual bird using the sum of all assigned femoral and tibial lesions, which ranged from 0 (absence of all lesions)―14 (most severe in both lesion types). A logistic regression analysis was conducted between all condition scores (femoral, tibial, body) and body weight data only in the Almaty and Akmola regions due to confounding factors that arose from the sourcing issues in the Abai region as briefly discussed above. All birds missing lesion scores and/or weight data were excluded from this analysis.

#### 2.3.3. Statistical Analyses

All data was initially entered and processed in Microsoft Excel (Microsoft Corporation, Redmond, WA, USA) followed by further processing in JMP Student Edition 19 (SAS Institute, Cary, NC, USA). Simple visualization of weight and lesion frequency was done using Microsoft Excel, while statistical analyses were conducted using JMP Student Edition 19. Weight comparison of birds between ages was evaluated using nonparametric Kruskal–Wallis rank sums test and followed by post hoc Dunn’s test. A logistic regression was applied to condition scores and body weight as previously described. All statistical significance was determined at *p* < 0.05.

## 3. Results

### 3.1. Weight Comparison

As expected, significant differences in bird body weight across ages were detected (Kruskal–Wallis *p* < 0.0001). Table 3 outlines broiler body weight data (including means ± standard deviation) and Kruskal–Wallis nonparametric mean comparison, followed by post hoc Dunn’s test.

### 3.2. Femoral and Tibial Lesions

**Figure 3:**
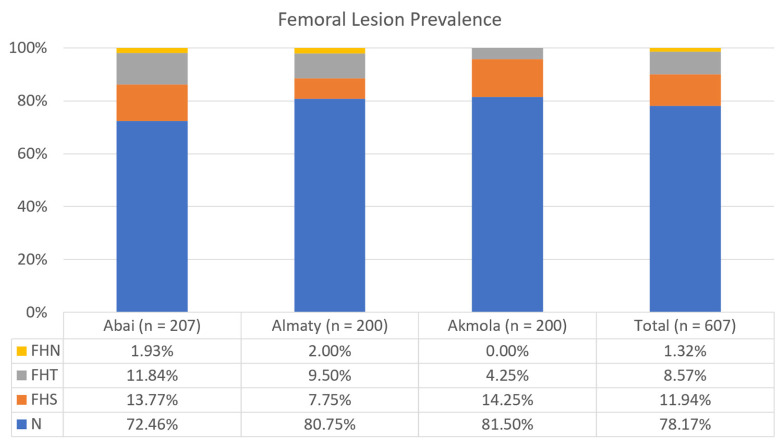
Distribution of femoral lesion severity categories. N = normal femoral head; FHS = proximal femoral head separation; FHT = proximal femoral head transitional degeneration; and FHN = proximal femoral head necrosis.

**Figure 4:**
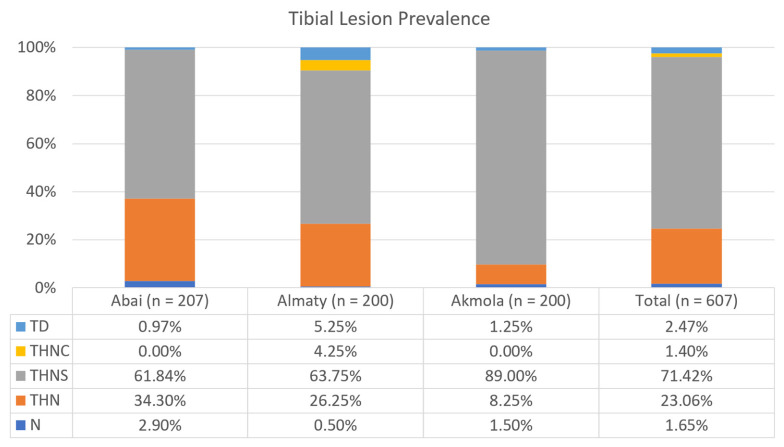
Distribution of tibial lesion severity categories. N = Normal Tibial Head; THN = Proximal Tibial Head Necrosis; THNc = Proximal Tibial Head Necrosis Caseous; THNs = Proximal Tibial Head Necrosis Severe; TD = Tibial Dyschondroplasia (non-bacterial disorder).

**Figure 5:**
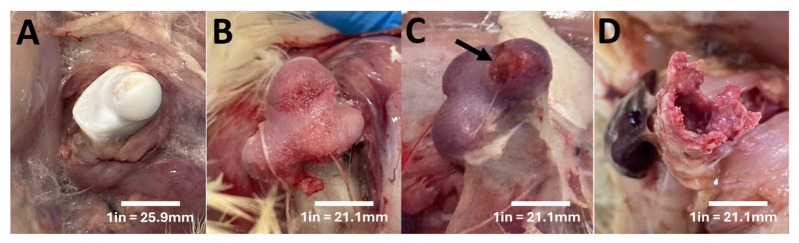
Field examples of proximal femoral lesions found in broilers, from left to right: (**A**): normal femoral head (N) with intact articular cartilage; (**B**): femoral head separation (FHS) with exposed, smooth epiphyseal head; (**C**): femoral head transitional degeneration (FHT) with moderate damage of exposed epiphyseal head (arrow); (**D**): femoral head necrosis (FHN) with complete fracturing of the epiphysis. Illustrated scale bars in all examples are fixed at a length of 25.4 mm (1 inch) with intended real lengths included per individual example.

**Figure 6:**
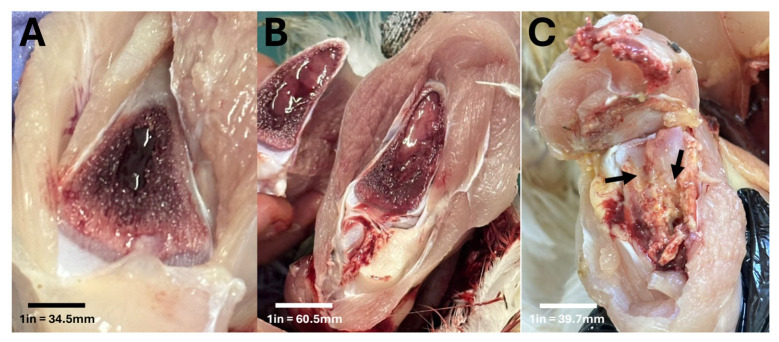
Field examples of proximal tibial lesions found in broilers, from left to right: (**A**): tibial head necrosis (THN) with small necrotic void; (**B**): severe tibial head necrosis (THNS) with large necrotic void encroaching on growth plate (physis); (**C**): caseous tibial head necrosis (THNC) with extensive necrosis and presence of “cheese-like” caseous exudate indicating bacteria foci (arrows). Illustrated scale bars in all examples are fixed at a length of 25.4 mm (1 inch) with intended real lengths included per individual example.

The distribution of femoral and tibial lesions, regardless of age, in all surveyed regions and in total is presented descriptively in Figure 3 and Figure 4, respectively. Likewise, photographed examples of each lesion type are also presented in Figure 5 and Figure 6, respectively.

Overall, in terms of femoral lesions, all regions saw similar distribution of lesion severity categories resembling overall distribution trends, with the majority having no damage (78.17%). On the other hand, with tibial lesions, broilers from the Akmola region had the markedly highest distribution of severe tibial head necrosis (89%). Remarkably, very few birds retained normal tibial head physiology in all regions (1.65%)―even in the youngest age group (d22; 14/17 cases with THN and/or THNS in both tibiae).

### 3.3. Body Weight and Condition Regression Analysis

As described, a logistic regression fit was applied to broiler weight data and condition scores (femur, tibia, body) in the Akmola and Almaty regions. No significant correlation was found between these parameters in either region.

## 4. Discussion

Live weight data taken from sampled broilers in surveyed Kazakh regions are comparable to those observed in Western regions at market age―particularly in the U.S. (average of 2.9 kg) [21]. However, data on broiler body weight across the surveyed Kazakhstani regions (Table 3) reveal significant variability between sites. This suggests that management and environmental factors, alongside bird age, likely play a major role. For instance, in Abai-1, average weights ranged from 0.786 ± 0.440 kg at 22 days to 3.042 ± 0.514 kg at 45 days (dropping slightly to 2.736 ± 0.529 kg by day 60). In contrast, Abai-2 recorded 2.495 ± 0.349 kg at 40 days. Weights at the farm in the Akmola region stood at 2.114 ± 0.264 kg at 46 days, while the Almaty site showed 1.817 ± 0.355 kg at 51 days. These inter-site differences were statistically significant (*p* < 0.05). Overall, while these values fall within the broad range previously reported for Kazakhstan’s poultry sector by the FAO [22], some sites clearly lagged in growth despite the birds being older. Based on field observations, this lower body weight is likely tied to a combination of feeding practices and microclimate challenges during the cold season. Although most farms followed a standard starter–grower–finisher schedule, their feed sources and finishing phase practices differed. Abai-2 used a plain commercial feed, whereas Abai-1 and Almaty supplemented their commercial finisher with crushed corn; meanwhile, the Astana site relied on its own in-house production. Without uniform data on nutrient density or specific formulas, we cannot rule out how variations in feed quality or mixing stability might have affected these growth rates.

Other environmental stressors might have also played an important role in observed suboptimal bird growth. After the brooding period, birds were moved to floor housing where temperatures often dropped below recommended levels. In the cold season, such conditions likely forced birds to divert energy toward thermoregulation instead of growth. At the same time, the presence of an underfloor heating system in Abai-1 likely contributed to maintaining a lower relative humidity (35–45%) compared to Abai-2 (55–70%). Such differences in the microclimate are of fundamental importance, since elevated humidity and damp bedding create conditions for an increased bacterial load and, consequently, an increased risk of lameness, which may explain the differences in the severity of lesions even within a single region. Moreover, high humidity (55–70%) and damp litter at Abai-2 and Almaty locations also possibly further compounded such stress. As reported, growth patterns were not uniform across locations, suggesting that management and environmental factors play a critical role in achieving optimal body weight under commercial conditions. While poultry operations in the Republic of Kazakhstan employ an extensive range of housing systems appropriate for different climatic conditions and physical levels in birds [23], proper management from farmers remains an indispensable role in upkeep of broiler health, welfare, and productivity. Ultimately, these regional disparities likely reflect inconsistent management―specifically regarding nutrition, temperature control, and litter quality―and should be viewed within the study’s limits, such as the uneven availability of feed data and lack of standardized climate monitoring. Therefore, presented data highlights the importance in consideration of regional production conditions when evaluating broiler performance in future studies.

As presented in Figure 3 and Figure 4, there are clear trends in terms of proximal femoral and tibial head health in all three regions―and compared to the average. Overall, the majority of broilers surveyed still retained normal proximal femoral heads, with intact articular cartilage caps (78.18%). This is in great contrast with trends observed in broilers reared under experimental BCO induction, where various aspects related to nutrition and housing environment are deliberately designed to be highly challenging to the animals [7,9]. Regardless, a moderate degree of FHS and FHT prevalence was observed in all four regions, signifying presence of BCO pathology in birds. It is important to note that, despite this study’s focus on subclinical BCO lesions, severe forms of THN and THNS are closely associated with clinical lameness: field observations have shown that birds with high lesion scores exhibited reduced mobility and altered gait, confirming their significance as indicators of well-being and the functional status of the musculoskeletal system. On the other hand, damage to the tibial head was extensively observed in all surveyed regions, with severe necrosis (THNS) making up the majority of cases (71.42%), particularly in the Akmola region (89%). As briefly mentioned, cases of THN and THNS were almost ubiquitous among necropsied broilers, even those at a young age―where clinical lameness cases and lesions are typically low [8,9,24].

With regard to the proximal femoral head, the general lack of damage and lesions can most likely be attributed to lighter average body weight of surveyed birds compared to those in a research setting, whose average weight can reach up to 4.53 kg by the eighth week of life [7]. As typical in research investigating BCO lameness and mitigatory strategies, a common goal is the coupling of sustained rapid growth rate, which causes cumulative mechanical damage to “prime” the bone for subsequent infection, and exposure to etiological agents generally associated with bone damage [6]. This is achieved by a combination of etiological spread (whether artificially challenged [9] or via mechanical induction [25,26]), sustained high activity levels through exposure to an extended photoperiod for the entire experimental period [26], and exacerbated by heavy weight from *ad libitum* feeding, which has been noted in literature [25,27]. When combined, these experimental conditions often manifest in the state of the femoral head, particularly by the presence of the articular cartilage cap in subclinical diagnosis. Upon necropsy and disarticulation of the femoral head, the cartilage can present as intact (normal) or readily retained in the acetabulum. In the latter, the epiphysis may readily separate with little to no damage (separation), or moderate damage (transitional), or be fractured from weakened necrotic structure (necrosis). As also noted by Wideman Jr et al. [25], this phenomenon is observed unilaterally and almost at the same frequency immediately post-necropsy, regardless of the force applied to femoral disarticulation, indicating its reliability as a diagnostic sign. Simply put, in the absence of stressful factors commonly seen in BCO research, standard practices in the poultry industry are unlikely to result in extensive femoral damage―particularly with lighter average broiler body weights―which may explain the moderately low prevalence of femoral head lesions in this study.

In contrast, the almost-ubiquitous presence of BCO lesions in the proximal tibial head across all regions and bird ages may imply more concerning issues. The prevalence of significant tibial pathology in the presence of a relatively intact femur may be attributed to the specific mechanical stress patterns in modern broiler breeds: the proximal tibia, which has a more active growth zone, is more susceptible to microdamage and subsequent bacterial colonization during the early stages of intensive growth. A possible underlying cause may be increased on-farm bacterial exposure, which has been previously reported by Zikibayeva, et al. [19] where bacterial infection of species often associated with BCO lameness―including *Salmonella* spp., *Escherichia coli*, *Clostridium* spp.―was ranked by local farmers as the most frequently recurring disease in participating regions. In the same article, the authors reported that even though basic biosecurity measures are practiced in both small and large-scale poultry farms, inadequate sanitary downtime between flocks and a general lack of “all in, all out” production cycles [28], which often leads to flocks with mixed ages, continue to be significant challenges in the effective control of infectious diseases [19]. As proper upkeep of biosecurity is the most important factor to safeguard bird health and productivity, it behooves local farmers and producers to strive for implementation of better production practices in order to reduce recurring disease spread, including infectious BCO lameness. Additionally, while recent plans for extensive expansion in the Kazakh poultry industry have painted bright outlooks for the future with self-sufficiency on the horizon [17], reliance on costly imports of production essentials (such as vitamin premixes, probiotics, and additives) may still impact production idiosyncrasies from local farmers, who may struggle to reach reasonable profits with further expenditures on additional supplements [29]. Considering the documented beneficial effects of such additive usage against BCO lameness [8,30,31], however, this aspect may warrant future considerations from farmers and producers―especially when optimal cost-effective practices have been consistently evaluated in the current literature [7,24].

Finally, as part of an updated protocol for BCO lameness survey in various global regions, we attempted to investigate whether a meaningful correlation between bone/body condition and collected weight data exists, using regression analysis. Our initial rationale was based on the currently accepted postulation of BCO pathogenesis, where rapid broiler body weight gain results in detrimental microfractures of immature leg bones and leads to subsequent bacterial infection [6]. However, as lameness worsens and causes increased pain to the animal, this affects productivity through lowered feed intake, weight gain, and feed conversion ratio. While there was no significant correlation found in the Almaty and Akmola regions, there is currently―to our knowledge―no comparative dataset of the same nature; thus, no concrete conclusion can yet be made regarding this analysis in a broader research scope. Additional data collection is therefore warranted for a complete picture regarding these parameters.

## 5. Conclusions

The survey of subclinical BCO lesions in three Kazakh regions (Abai, Akmola, and Almaty) revealed clear trends in terms of proximal and tibial head damage in broilers. Compared to broilers in a BCO research setting, broilers from Kazakh regions had a lower distribution of femoral head damage (78.17% Normal), but high distribution of severe tibial head necrosis (71.42%)―particularly in Akmola (89%)―despite a lower average broiler body weight. Additionally, average broiler weights showed inconsistent trends both between regions and with expected age. These findings may imply significant exposure to etiological agents associated with BCO subclinical lesions and lameness from suboptimal production management, which warrants further future investigation to develop effective mitigatory and preventive strategies against the disease in these regions.

## Figures and Tables

**Table 1:** Detailed sampling location information.

Region	Survey Date	Breed	Housing Type	Bird Age (Days)	Number of Animals
Abai-1	November 2025	Arbor Acres	Floor housing with wood shavings	22, 45, 60	22 d = 17 45 d = 27 60 d = 64
Abai-2	December 2025	Arbor Acres	Floor housing (5 days on straw followed by wood shavings)	40	100
Almaty	December 2025	Arbor Acres	Floor housing with wood shavings	51	200
Akmola	January 2026	Cobb 500	Floor housing (5 days on straw followed by wood shavings	46	200

**Table 2:** Numerical lesion scoring system.

Score	Femoral Lesion Severity	Tibial Lesion Severity
0	N (Normal)	N (Normal)
1	FHS (Femoral Head Separation)	THN (Tibial Head Necrosis)
2	FHT (Femoral Head Transitional)	THNs (Tibial Head Necrosis Severe)
3	FHN (Femoral Head Necrosis)	THNc (Tibial Head Necrosis Caseous)
4	N/A	TD (Tibial Dyschondroplasia)

**Table 3:** Nonparametric comparison of broiler weight by age.

Broiler Age (Day)	Region	Number of Animals (n)	Weight Means (kg) ± SD
22	Abai-1	17	0.786 ± 0.440 ^a^
40	Abai-2	100	2.495 ± 0.349 ^b^
45	Abai-1	27	3.042 ± 0.514 ^b^
46	Akmola	200	2.114 ± 0.264 ^c^
51	Almaty	200	1.817 ± 0.355 ^d^
60	Abai-1	64	2.736 ± 0.529 ^b^

Note: Non-connecting superscript letters denote significant statistical difference (*p* < 0.05).

## Data Availability

All original contributions presented in this study are included in the article. Raw data can be obtained from corresponding authors upon reasonable request.

## Abbreviation

The following abbreviation is used in this manuscript:

BCO	Bacterial Chondronecrosis with Osteomyelitis
